# Reuse of the Materials Recycled from Renewable Resources in the Civil Engineering: Status, Achievements and Government’s Initiatives in Taiwan

**DOI:** 10.3390/ma14133730

**Published:** 2021-07-02

**Authors:** Chi-Hung Tsai, Yun-Hwei Shen, Wen-Tien Tsai

**Affiliations:** 1 Department of Resources Engineering, National Cheng Kung University, Tainan 701, Taiwan; ap29fp@gmail.com; 2 Graduate Institute of Bioresources, National Pingtung University of Science and Technology, Pingtung 912, Taiwan

**Keywords:** industrial waste, renewable resource, reuse, civil engineering material, regulatory promotion

## Abstract

Growing concerns about the circular economy and sustainable waste management for civil applications of non-hazardous mineral industrial waste have increased in recent years. Therefore, this study presents a trend analysis of industrial waste generation and treatment during the years of 2010–2020, and focused on promotion policies and regulatory measures for mandatory renewable resources from industrial sources in Taiwan, including reclaimed asphalt pavement (RAP) material, water-quenched blast furnace slag, and ilmenite chlorination furnace slag. According to the official database of the online reported statistics during the period of 2010–2020, approximately three million metric tons per year of renewable resources were totally reused in civil engineering or related cement products, reflecting a balanced supply chain in the domestic market. Among these, water-quenched blast furnace slag accounted for about 90% (about 2.7 million metric tons) in Taiwan. Currently, the legislative framework of sustainable waste management in Taiwan is based on the Waste Management Act and the Resource Recycling Act, but there are some problems with them. In order to effectively reduce environmental loadings and conserve natural resources to mitigate climate change, some recommendations are addressed from different points of view.

## 1. Introduction

Situated to the east of mainland China, and as an export-dependent economy of 23.4 million people, Taiwan has shown rapid industrial development over the past few decades and now occupies a strategic position in global supply chains, such as those of microchips and information and communication technology (ICT) products. However, the enormous economic development in Taiwan has resulted in a greater complexity of industrial waste management during this period. Before the Taiwan government revised the Waste Management Act in 1999, businesses either had to dispose of their waste on their own or commission government departments. At that time, industrial waste was often abandoned illegally and/or exported to other Asian countries due to incompetent government administrations, insufficient capacity of waste treatment facilities, and the high cost of hazardous waste treatment [1,2,3]. In order to efficiently reduce waste generation and also follow international trends in a hierarchy of waste management [4,5], the Taiwan Environmental Protection Administration (EPA) revised the Act in 1999, and formed a legal frameworks for industrial waste management, including online reporting, strict penalties and treatment methods [6,7]. Since 2000, the EPA has formulated the “National Industrial Waste Management Program” and the “Industrial Waste Control Center”, as well as a tracking system that controls the life cycle of waste from generation to disposal [8]. These historical developments of industrial waste management in Taiwan were similar to those of other Asian countries, such as Japan [9], Korea [10], China [11], and India [12].

In order to significantly reduce environmental loading and effectively reuse available materials from waste resources for the targets of zero waste [13,14,15] and industrial and urban symbiosis [16], the EPA has been actively undertaking the 4-in-1 Recycling Program under the authorization of the Waste Management Act since the early 2000s. This program integrates community residents, responsible and recycling enterprises, local governments, and a non-profit recycling fund for the recycling of regulated recyclable wastes among municipal solid wastes, especially in electrical and electronic waste equipment (WEEE) [17]. On the other hand, the EPA newly enacted the Resource Recycling Act in 2002 [18], which aims at conserving natural resources, promoting the recycling and reuse of materials, and mitigating environmental loading. The act focused on promotional policies and measures on the recycling and reuse of renewable resources from industrial waste sources, which were announced by a competent central industry authority. In this regard, reclaimed asphalt pavement (RAP) material has been listed as a renewable resource by the Ministry of Interior (MOI) because it has been reused as raw material for asphalt concrete since the 1990s [19,20,21]. In addition, furnace slags, including quenched blast furnace slag and ilmenite chlorination furnace slag, have been listed as renewable resources by the Ministry of Economic Affairs (MOEA) due to their successful application in cement products of Taiwan [22,23,24] and other regions [25].

As reviewed above, it was seldom found in combination with a description of the current status and regulatory measures of renewable resources from industrial waste in Taiwan’s engineering applications. Therefore, this paper firstly analyzed the trends of industrial waste generation and treatment during the period of 2010–2020; furthermore, an updated status of the announced renewable resources that can be reused in civil engineering was also discussed in the study. Finally, regulatory promotion of renewable resource reused in civil engineering was addressed to echo a case study in the establishment of Environmental Science and Technology Parks (ESTP).

## 2. Data Mining

The main purposes of this study were to analyze the updated status of industrial waste and announced renewable resources (i.e., reclaimed asphalt pavement material, water-quenched blast furnace slag, and ilmenite chlorination furnace slag), and further address regulatory measures for promoting them in civil engineering applications. Therefore, a statistical database, recycling and reuse, and regulatory measures relevant to the announced renewable resources are briefly summarized below.

- Activity (statistics and status) of industrial waste generation and treatment:

The updated data on the statistics and status of industrial waste generation and treatment in Taiwan were obtained from the official yearbook [26] and website [27], which were compiled by the EPA.

- Activity (statistics and status) of renewable resources reused in civil engineering:

In order to highlight the recycling and reuse of renewable resource in Taiwan, the updated data on the statistics and status of the announced items (i.e., reclaimed asphalt pavement material, water-quenched blast furnace slag, and ilmenite chlorination furnace slag) reused in civil engineering were also accessed on the official website [27].

- Regulatory measures for the renewable resources and the establishment of ESTP:

Information about the regulatory measures for the announced renewable resources was accessed on the relevant website [28]. In addition, an official plan for the establishment of ESTP was addressed to echo the regulatory promotion for the recycling and reuse of renewable resources [29].

## 3. Results and Discussion

### 3.1. Trend Analysis of Industrial Waste Generation and Treatment

#### 3.1.1. Industrial Waste Generation

Based on the Waste Management Act in Taiwan, there are two categories of industrial waste (i.e., general industrial waste and hazardous industrial waste), which refer to waste generated from industry activities, but excludes waste generated by the employees of those industries themselves. Herein, the industry activities include agricultural, industrial and mining plants and sites, construction enterprises, medical/hospital/clinic organizations, public and private waste management (clearance, treatment, and disposal) organizations, joint industrial waste treatment organizations, laboratories of schools or agency groups, and other enterprises designated by the central competent authority. General (non-hazardous) industrial waste is composed of movable solid, liquid substances, or objects other than hazardous industrial waste. In contrast, hazardous industrial waste is legally identified by its toxic characteristics and hazardous substances due to their negative impacts on human health and the environment when it is not managed properly. The central competent authority as referred to in the act means the EPA. It should be noted that radioactive waste management is in accordance with the relevant atomic energy regulations, such as the Ionization Radiation Protection Act.

**Figure 1:**
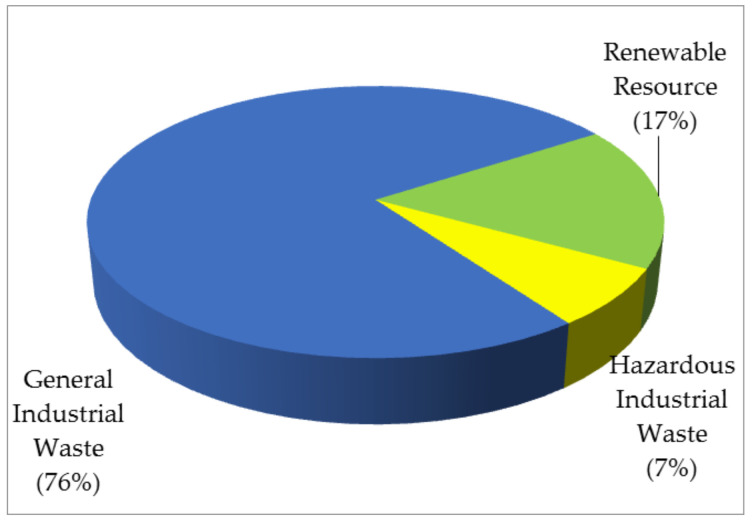
Pie chart of generation percentages of three categories of industrial waste in 2019.

In July 1999, the act was revised to promulgate a legal framework for industrial waste management, thus formulating the “National Industrial Waste Management Program”, the “Industrial Waste Control Center”, and the online reporting system that has tracked the complete life cycle of industrial wastes since 2001 from the generation source to the final disposal. On the other hand, the Resource Recycling Act was newly enacted to further promote recycling and reuse of so-called renewable resource, which are produced or derived from general industrial waste. Currently, there are three renewable resources announced by the central industry authorities, reclaimed asphalt pavement material, quenched blast furnace slag, and ilmenite chlorination furnace slag. Table 1 lists the reported amounts of industrial waste generation during the decade of 2010–2020 in Taiwan by accessing the official database [27]. Figure 1 shows the generation percentages of general industrial waste, hazardous industrial waste, and renewable resource, in 2019. Obviously, the reported amounts indicated a slight increase from about 18.1 million metric tons in 2010 to a peak record of approximately 22.3 million metric tons in 2018. Thereafter, the reported amounts showed a decreasing trend from 2019. The significant decrease during the years of 2018–2019 can be attributable to the exclusion of waste generated by the employees themselves (since 2019). As compared to the amounts of industrial waste generation in 2005 (i.e., about 14.6 million metric tons), it reflects the ongoing implementation of industrial waste minimization (including source reduction and waste recycling and reuse) in the industrial sector since the early 2000s [26,27], thus retarding the expected increase during the decade. For example, Taiwan’s semiconductor industries contributed significantly to the supply chain globally, but also generated large amounts of industrial waste, such as calcium fluoride (CaF_2_) sludge. Currently, this sludge is reused as raw cement material in Taiwan [28].

#### 3.1.2. Industrial Waste Treatment

**Figure 2:**
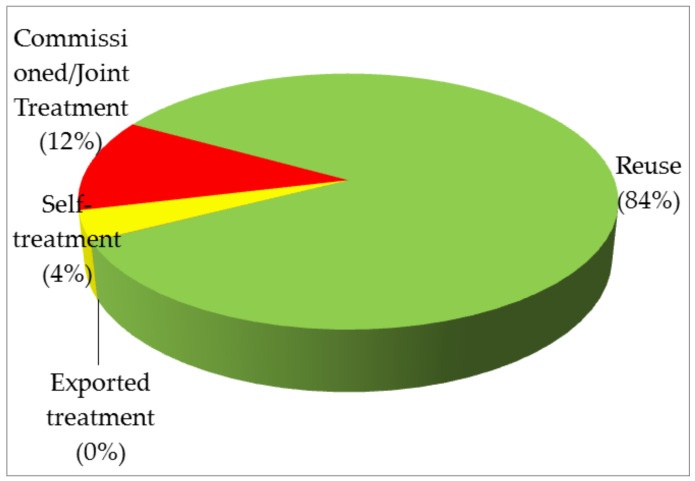
Pie chart of percentages of four methods of industrial waste treatment in 2020.

As described above, industrial waste management in Taiwan started with the implementation of the Waste Management Act, which has been revised several times. According to the official definition of industrial waste treatment, the term “treatment” refers to three methods, immediate treatment (e.g., incineration, solidification), final disposal (e.g., sanitary landfill), and reuse. The term “reuse” is defined as “the reuse of industrial waste produced by an enterprise as raw material, materials, fuel, land reclamation fill, or other reuse methods recognized by the central industry competent authority via self-use, sale, transfer, or commissioning, and in compliance with the related regulations”. Table 2 summarizes the reported amounts of industrial waste treatment during the period of 2010–2020 [26,27]. Figure 2 depicts the percentages of four methods of industrial waste treatment in 2020. Obviously, under the circular economy principle, focusing on waste recycling, the amounts of reused industrial waste indicated an increasing trend over the past decade, reaching an industrial waste reuse and recycling rate of over 80%. In fact, the EPA amended the Waste Management Act on 24 October 2001 to promote proper industrial waste management and reuse, leading to a higher willingness to reuse industrial waste and resource materials. Article 39 mandates that industrial waste reuse be implemented as required by the central industry competent authorities. Since then, there have been 10 agencies for formulating the corresponding regulations and management systems, which are relevant to industrial waste reuse under their jurisdictions.

### 3.2. Status of Renewable Resource Reused in the Civil Engineering

**Figure 3:**
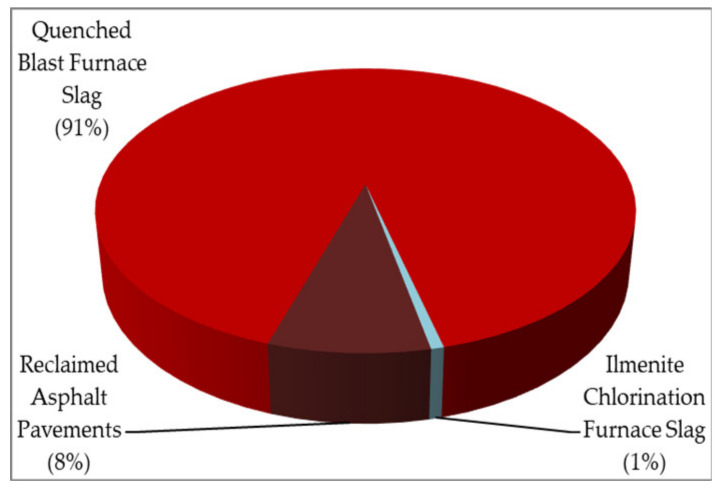
Pie chart of percentages of three categories of renewable resources in 2020.

Under the authorization of the Resource Recycling Act, a so-called “renewable resource” is defined as “the announced substances that have lost their original usefulness, are economically and technologically feasible to recycle, and may be recycled or reused” [28]. Herein, the term “reuse” means the practice of making direct and repeated use of renewable resources in their original form or using renewable resources after restoring some or all of their original functionalities. Another term, “recycling”, means the practice of making renewable resources functional by altering the original form of substances, or combining them with other substances, so that they may serve as materials, fuel, fertilizers, animal feed, fillers, soil enhancers, or for other uses recognized by the central industry authorities. Regarding the differences between the terms reuse and recycling, they obviously have certain overlapping and ambiguous scopes, but reuse should have a preferred hierarchy. According to the “renewable resource” lists announced by the central competent authorities (i.e., MOEA, MOI, and EPA), they include water-quenched blast furnace slag, ilmenite chlorination furnace slag, cobalt-manganese compound precipitate, scrap masonry material, reclaimed asphalt pavement material, and electronic waste-derived materials (iron, copper, aluminum, glass, and plastic). Obviously, slag materials are typically referred to as industrial “by-products”. These mining resources, once used to produce cement, are not renewable and circular since they are not used in the production of either fuel or cement again in the future. In this regard, the definition of “renewable resource” should be amended in the Resource Recycling Act. Table 3 summarizes the reported amounts of renewable resource generation during the period of 2010–2020 [26,27]. Figure 3 indicated the percentages of three categories of renewable resources in 2020, the majority being water-quenched blast furnace slag. As shown in Table 3, approximately 3 million metric tons of renewable resources were reused annually in civil engineering or in related cement products, reflecting a balanced supply chain in the domestic market. These announced renewable resources were completely reused or recycled in civil engineering or related products, such as cement materials, which will be further addressed in Section 3.3.

In fact, there are many different slags from manufacturing industries in Taiwan, including electric arc furnace steelmaking, induction furnace steelmaking, cupola furnace steelmaking, water-quenched blast furnace steelmaking, basic oxygen furnace (BOF) steelmaking, ilmenite chlorination furnace titanium dioxide (TiO_2_)-making, and rotary kiln in the thermal reuse treatment of steelmaking dust. Currently, slags from water-quenched blast furnace steelmaking and ilmenite chlorination furnace TiO_2_-making have been listed as “renewable resources” by the central industry competent authority (i.e., MOEA). In Taiwan, water-quenched blast slag powder (after being ground) is blended with cement to replace general cement and is widely applied in various kinds of construction projects. On the other hand, RAP comes from oil-based tar (a fossil resource). RAP materials were derived from frequent milling and overlay activities for roads, and thus are produced in considerable amounts annually. In Taiwan, experience has indicated that the recycling of bitumen binder in RAP is a circular economy approach from technical, economical, and environmental viewpoints [19,20,21]. Therefore, RAP has been listed as one of the “renewable resources” by the central industry competent authority (i.e., MOI). In this regard, the Implementation Guidelines for the Outline Specifications for Public Construction—Chapter 02966 in Taiwan has allowed incorporating, at most, 40% of RAP into new hot-mix asphalt (HMA) since the late 1990s [21].

### 3.3. Regulatory Promotion for Renewable Resource Reused in the Civil Engineering

In Taiwan, the government established a renewable resource recycling system with the enactment of the Waste Management Act (also called the Waste Disposal Act) and the Resource Recycling Act [28]. The central industry authorities (i.e., MOEA and MOI) should be in consultation with the central competent authority (i.e., EPA) for announcing renewable resource items that must be recycled or reused according to the promulgated measures.

#### 3.3.1. Waste Management Act

In Taiwan, industrial waste management started with the promulgation of the Waste Management Act in 1974, which has been revised several times to comply with the domestic industrial structure and international trends. When established in 1987, the EPA began to put emphasis on industrial waste minimization (or cleaner production) and also set up a permit system concerning waste generation sources and waste treatment organizations. In recent years, the waste management concept has shifted to source reduction approaches, such as circular economy, sustainable materials management (SMM) and cradle-to-cradle (C2C). In order to promote industrial waste recycling and reuse, the regulatory measures and incentives have been incorporated into the act, which will be briefly described below.

The disposal of industrial waste, with the exception of that subject to reuse methods, shall be performed in accordance with the following methods:

1. Any product from industry activities shall be determined as waste when it is under any of the following circumstances:
  - The product is determined to be of no economic or market value by the EPA, and is intended to be disposed of illegally or harmful to the environment and human health.
  - The product is not lawfully stored or used, and is intended to be disposed of illegally or causing pollution.
  - The recycled/reused product is not used in accordance with this act, and is intended to be disposed of illegally or causing pollution.
2. Industrial waste management, with the exception of that subject to recycling/reuse methods, shall be performed in accordance with the methods of self-treatment, joint treatment and commissioned treatment.
3. Industrial waste reuse shall be processed in accordance with the regulations promulgated by the central industry competent authorities or central competent authority. As listed in Table 2, most industrial waste is currently reused or recycled as materials, fuel, land reclamation fill, and soil modifier according to the corresponding regulations by the ten authorities, especially in the Ministry of Economic Affairs (MOEA), the Council of Agriculture (COA), and the Ministry of Interior (MOI).
4. The expenses incurred by the enterprise to waste management should be partially exempted from tax. Furthermore, the enterprises that are in compliance with relevant waste management regulations and with excellent performance in the waste reduction, recycling, and reuse shall be rewarded by the EPA and the central industry competent authority.

#### 3.3.2. Resource Recycling Act

In order to conserve natural resources, reduce waste generation, promote recycling and reuse of materials, and mitigate environmental loading, the Resource Recycling Act was passed on 21 January 2002. The act stipulates that businesses or enterprises must be in accordance with the management regulations when recycling or reusing the designated renewable resources designated by the central industry authority. The current promotion (assistance and incentive) measures for recycling or reusing renewable resources in industry were briefly addressed as follows:

1. To promote the recycling and reuse of renewable resources, the public organizations shall preferentially procure the government-certified environment-friendly products, domestic renewable resource, or recycled products in which contain a certain proportion of renewable resource. Therefore, the EPA requested relevant government agencies to consider the possibility of using products made from recyclable and reusable waste materials in their public construction projects. Through the efforts of these government agencies, the usage of such products in public construction has been increased gradually. As listed in Table 3, the three renewable resources were totally reused or recycled in the civil engineering or related products like cement and filling materials. It should be noted that the products recycled or recused from the renewable resources must comply with the operation management requirements like the national standards, international standards, engineering specifications, and other relevant regulations. For example, ilmenite chlorination furnace slag must comply with the Chapter 02726 of Taiwan’s Construction Specifications (i.e., specific gravity ≥ 1.5, water absorption ≤ 25%, and immersion swelling ratio ≤ 0.5%) when it is reused as bottom grade granular material in the pavement works.
2. The EPA shall regularly hold the awards for excellence in recycling/reusing technological developments and their actual achievements. The business or enterprise engaged in renewable resource recycling and reusing shall be granted tax incentives (tax deduction) for the cost of investment in research, facilities, tools, and equipment. In this regard, the “Award Ceremony for Excellent Performance of Industrial Waste and Resource Recycling and Reuse” has been held annually. In addition, the EPA held symposiums and exhibitions annually to further share the successful recycling experience with the general public and the industrial sector.
3. To promote the recycling and reuse of renewable resources, acquire advanced technologies and talents, and encourage innovative research and development (R&D) technologies by the domestic industry, the EPA in collaboration with the local governments established the environmental science and technology parks [29], which will be further addressed in the next section as a case study.

Table 4 lists the items, sources, and reuse types of renewable resource announced by the central industry competent authority [28]. In this regard, reclaimed asphalt pavement (RAP) material is widely used as a cheaper alternative to the conventional hot mix asphalt (HMA), but the mixing ratio shall not exceed 40% according to the commonly used practice [30]. As shown in Table 3, about 3 million metric tons of renewable resources were reused annually in the civil engineering or related cement products.

### 3.4. Case Study: Establishment of Environmental Science and Technology Parks

Since the 1992 United Nations Conference on Environment and Development (UNCED) held in Rio de Janeiro (Brazil), sustainable development and recycling-oriented societies have become global trends. Thereafter, one of the significant policies was to establish ecological industrial zones by the developed countries for incorporating industrial development into the natural ecological system. In 2001, the EPA started planning the establishment of Environmental Science and Technology Parks (ESTP) for jointly cooperating with local governments. The three main development axes of ESTP are “high-level resource recycling technology”, “high-level environmental protection technology” and “ecological industries”. The ESTP proposal was approved by the Executive Yuan (the Cabinet) on 9 September 2002, and was revised on 11 March 2004. The total budget for the ESTP planning and construction has reached 6.2 billion NT$ (about 0.22 billion US$). In order to successfully develop the ESTP, the EPA and local governments cooperated to construct so-called sustainable ecological communities. In this regard, the EPA was responsible for providing financial incentives to attract enterprises which needed to be reviewed by the examination mechanism and administrative measures. In addition, the EPA also provided tenants and research institutes with rewards for investing in technology development and industrial production of environment-friendly products. By contrast, the local governments were responsible for providing sufficient and suitable land, and administrative services for establishment, recruitment and operations.

**Figure 4:**
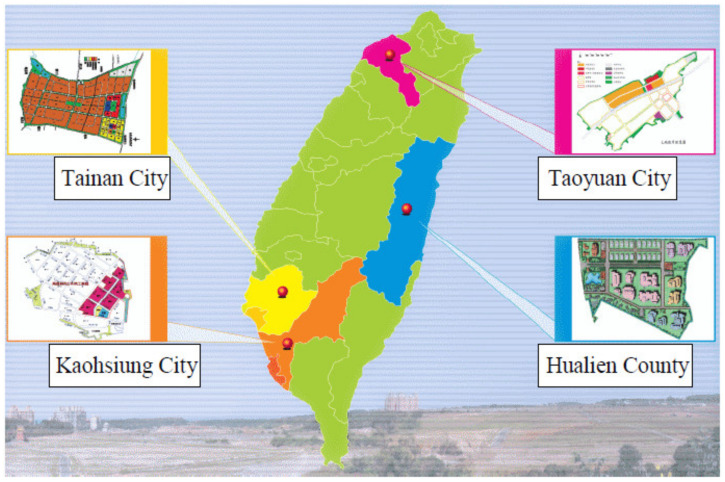
Locations of environmental science and technology parks in Taiwan [31].

By the end of 2011, the EPA had established four ESTP zones in Taiwan, as shown in Figure 4 [31]. They cover a total area of 123 acres; Benzhou ESTP (40 acres) in Kaohsiung City, Fenglin ESTP (22 acres) in Hualien County, Guanyin ESTP (31 acres) in Taoyuan City, and Liouying ESTP (30 acres) in Tainan City. In order to attractively promote recruitment, the government subsidized 50% of the land rent payment, and provided 25 million NT$ for total production and 50% of the investment in R&D. Currently, over 60 businesses have completed tenant procedures. It was estimated that 13.5 billion NT$ will be pooled from the private sector, and that these ESTP will create an annual revenue of about 3.0 billion NT$ (0.21 billion US$), in addition to over 2000 job opportunities and approximately 3 million metric tons of recyclable resources through mass production demonstration area in the ESTP. Furthermore, the recycling and reuse of renewable and recyclable resources from general waste (municipal solid waste) and industrial waste will form a complete green supply chain and a circular economy to assist businesses in lowering their operation costs and also achieve their corporate social responsibility (CSR) goals.

## 4. Conclusions and Recommendations

With the implementation of renewable resources recycling and reuse in Taiwan under the authorization of the Resource Recycling Act in 2002, reclaimed asphalt pavement material, water-quenched blast furnace slag, and ilmenite chlorination furnace slag have been listed as “mandatory” recyclables by the EPA for the production of recycled aggregate materials in concrete and construction applications. During the period of 2010–2020, there were approximately three million metric tons of renewable resources reused annually in civil engineering or related cement products, reflecting a balanced supply chain in the domestic market. Currently, these announced renewable resources are completely reused in civil engineering or related products like cement material. This approach, not only reduces the amount of industrial waste disposal, but also recycles the valuable resources. More significantly, this sustainable waste management approach also raised green productivity and drove supply chain sustainability to develop a circular economy.

Currently, the legislative framework of sustainable waste management in Taiwan is based on the Waste Management Act and the Resource Recycling Act. However, there are some problems between them, including unclear definitions (e.g., reuse/recycling, waste/discard/resource, renewable resource/by-product), waste generation sources and central competent authorities. In the near future, major sources of industrial waste in Taiwan will be generated from the high-tech industries due to the global supply chains. To further enhance the recycling and reuse of WEEE and mineral industrial waste, the following measures were recommended:

- Combining the Waste Management Act and the Resource Recycling Act into a new act, which will incorporate the 5R (i.e., reduction, reuse, recycling, recovery, and reclamation) principles towards the ultimate goal of zero waste through total recycling.
- Promulgating the specific regulations for high-tech industries (e.g., semiconductor and opto-electronics manufacturing) to conduct an industrial symbiosis through industrial waste recycling and cleaner production.
- Adding several mineral waste sources (legally identified as non-hazardous industrial waste) to the lists of renewable resources like electric arc furnace slag, induced current furnace slag, coal ash, and scrap masonry material, which can be reused as available materials in civil engineering.
- Providing sufficient economic and financial (tax) incentives in the accounting/cost system of enterprises or businesses based on the performances of sustainable goals (SDGs) or corporate social responsibility (CSR).

## Figures and Tables

**Table 1:** Reported amounts of industrial waste generation during the decade of 2010–2020 in Taiwan ^1^.

Year	General Industrial Waste	Hazardous Industrial Waste	Renewable Resource	Total
2010	1.375 × 10^7^	0.122 × 10^7^	0.312 × 10^7^	1.809 × 10^7^
2011	1.412 × 10^7^	0.120 × 10^7^	0.341 × 10^7^	1.873 × 10^7^
2012	1.392 × 10^7^	0.125 × 10^7^	0.278 × 10^7^	1.795 × 10^7^
2013	1.448 × 10^7^	0.145 × 10^7^	0.275 × 10^7^	1.867 × 10^7^
2014	1.424 × 10^7^	0.160 × 10^7^	0.300 × 10^7^	1.884 × 10^7^
2015	1.449 × 10^7^	0.137 × 10^7^	0.330 × 10^7^	1.916 × 10^7^
2016	1.420 × 10^7^	0.136 × 10^7^	0.342 × 10^7^	1.897 × 10^7^
2017	1.485 × 10^7^	0.144 × 10^7^	0.307 × 10^7^	1.937 × 10^7^
2018	1.774 × 10^7^	0.146 × 10^7^	0.313 × 10^7^	2.233 × 10^7^
2019	1.506 × 10^7^	0.139 × 10^7^	0.339 × 10^7^	1.984 × 10^7^
2020	1.680 × 10^7^		0.294 × 10^7^	1.975 × 10^7^

^1^ Sources [26,27]; unit: metric ton.

**Table 2:** Reported amounts of industrial waste treatment during the decade of 2010–2020 in Taiwan ^1^.

Year	Reuse	Self-Treatment	Commissioned or Joint Treatment	Exported Treatment	Total
2010	1.458 × 10^7^	4.943 × 10^5^	2.625 × 10^6^	3.329 × 10^4^	1.773 × 10^7^
2011	1.544 × 10^7^	4.988 × 10^5^	2.899 × 10^6^	3.119 × 10^4^	1.887 × 10^7^
2012	1.451 × 10^7^	5.007 × 10^5^	2.880 × 10^6^	3.160 × 10^4^	1.792 × 10^7^
2013	1.491 × 10^7^	8.124 × 10^5^	2.783 × 10^6^	5.077 × 10^4^	1.856 × 10^7^
2014	1.521 × 10^7^	8.658 × 10^5^	2.753 × 10^6^	4.950 × 10^4^	1.888 × 10^7^
2015	1.581 × 10^7^	6.099 × 10^5^	2.663 × 10^6^	4.665 × 10^4^	1.913 × 10^7^
2016	1.469 × 10^7^	6.358 × 10^5^	2.587 × 10^6^	1.582 × 10^4^	1.793 × 10^7^
2017	1.564 × 10^7^	6.547 × 10^5^	2.634 × 10^6^	1.544 × 10^4^	1.894 × 10^7^
2018	1.680 × 10^7^	6.889 × 10^5^	2.615 × 10^6^	0.771 × 10^4^	2.011 × 10^7^
2019	1.667 × 10^7^	7.129 × 10^5^	2.456 × 10^6^	0.824 × 10^4^	1.985 × 10^7^
2020	1.676 × 10^7^	7.442 × 10^5^	2.343 × 10^6^	0.882 × 10^4^	1.985 × 10^7^

^1^ Sources [26,27]; unit: metric ton.

**Table 3:** Reported amounts of renewable resource generation during the decade of 2010–2020 in Taiwan ^1^.

Year	Reclaimed Asphalt Pavement Material	Water-Quenched Blast Furnace Slag	Ilmenite Chlorination Furnace Slag	Total
2010	0.203 × 10^6^	2.739 × 10^6^	0.178 × 10^6^	3.120 × 10^6^
2011	0.273 × 10^6^	2.955 × 10^6^	0.183 × 10^6^	3.411 × 10^6^
2012	0.146 × 10^6^	2.590 × 10^6^	0.041 × 10^6^	2.777 × 10^6^
2013	0.135 × 10^6^	2.615 × 10^6^	- ^2^	2.750 × 10^6^
2014	0.257 × 10^6^	2.739 × 10^6^	- ^2^	2.996 × 10^6^
2015	0.351 × 10^6^	2.28 × 10^6^	0.017 × 10^6^	3.296 × 10^6^
2016	0.364 × 10^6^	3.034 × 10^6^	0.022 × 10^6^	3.420 × 10^6^
2017	0.429 × 10^6^	2.619 × 10^6^	0.026 × 10^6^	3.074 × 10^6^
2018	0.399 × 10^6^	2.702 × 10^6^	0.027 × 10^6^	3.128 × 10^6^
2019	0.330 × 10^6^	3.045 × 10^6^	0.014 × 10^6^	3.389 × 10^6^
2020	0.239 × 10^6^	2.685 × 10^6^	0.019 × 10^6^	2.943 × 10^6^

^1^ Sources [26,27]; unit: metric ton. ^2^ Not reported.

**Table 4:** Items and reuse types of renewable resource announced by the central industry competent authority.

Central Industry Competent Authority	Item	Definition by Generation Source	Reuse Type
Environmental Protection Administration (EPA) ^1^	Iron	Electronic waste (waste electrical and electronic equipment, WEEE) ^4^	Raw material for steel making, ferric chloride, or reused to related chemical products
	Copper	WEEE	Raw material for copper/steel products, or reused to its chemical feedstock
	Aluminum	WEEE	Raw material for aluminum products, or reused to its chemical feedstock
	Glass	WEEE (without containing fluorescent powder or liquid crystal)	Raw material for glass/ceramic tile/cement products, glass, cement; additive for concrete/asphalt concrete; or reused to its chemical feedstock
	Plastic	Electronic waste (or waste electrical and electronic equipment) ^4^	Raw material for plastic products and plastic pyrolysis; auxiliary fuel for cement/steel plants
Ministry of Interior (MOI) ^2^	Reclaimed asphalt pavement material	By-product of construction project for asphalt concrete excavation	Raw material for asphalt concrete; or engineering filling material(note: when reusing as a hot-mix recycled asphalt concrete, the mixing ratio shall not exceed 40%)
Ministry of Economic Affairs (MOEA) ^3^	Water-quenched blast furnace slag	By-product of steelmaking in wholly integrated steel mills where water-quenched blast furnace slag is formed by cooling slag in water	Raw material for quenched blast furnace slag powder, cement, cement products, ceramics, or fertilizer; concrete cementing material
	Ilmenite chlorination furnace slag	By-product of manufacturing titanium dioxide (TiO_2_) in the ilmenite chlorination process	Raw material for recycled aggregate (for base or bottom grade granular material in the pavement works, controlled low-strength material, cement products, base filling material, embankment filling material only), or cement products
	Cobalt-manganese (Co/Mn) compound precipitate (content of Co ≥ 10 wt%)	By-product of manufacturing pure terephthalic acid (PTA) and isophthalic acid (ITA)	Raw material for cobalt-manganese catalyst
	Scrap masonry material	By-product of stone products manufacturing	Raw material for remade stone (board, brick or block), tile, ceramic clay powder, premixed concrete, cement, cement products, construction material, limestone (for marble trims only), recycled aggregate, fertilizer (for serpentine trims only) and craft; controlled low-strength material (CLSM); material for horticultural landscaping

^1^ Promulgated on 1 December 2006. ^2^ Promulgated on 23 April 2007. ^3^ Promulgated on 16 January 2004 (quenched blast furnace slag and ilmenite chlorination furnace slag) and 11 September 2020 (cobalt–manganese compound precipitate and scrap masonry material). ^4^ Registered WEEE according to the Article 18 of the Waste Management Act.

## Data Availability

Not applicable.

## References

[1] Houng H.J. (1999). Industrial waste management in Taiwan. Environ. Pract..

[2] Wei M.S., Huang K.H. (2001). Recycling and reuse of industrial wastes in Taiwan. Waste Manag..

[3] Hsing H.J., Wang F.K., Chiang P.C., Yang W.F. (2004). Hazardous wastes transboundary movement management: A case study in Taiwan. Resour. Conserv. Recycl..

[4] Sakai S., Sawell S.E., Chandler A.J., Eighmy T.T., Kasson D.S., Vehlow J., van der Sloot H.A., Hartien J., Hjelmar O. (1996). World trends in municipal solid waste management. Waste Manag..

[5] Wilson D.C. (1996). Stick or carrot?: The use of policy measures to move waste management up the hierarchy. Waste Manag. Res..

[6] Tsai W.T., Chou Y.H. (2004). A review of environmental and economic regulations for promoting industrial waste recycling in Taiwan. Waste Manag..

[7] Tsai W.T., Chou Y.H. (2004). Government policies for encouraging industrial waste reuse and pollution prevention in Taiwan. J. Clean. Prod..

[8] Houng H., Cheng Y.W. (2013). Electronic tracking and management of industrial waste in Taiwan. J. Mater. Cycles Waste Manag..

[9] Tanaka M. (1999). Recent trends in recycling activities and waste management in Japan. J. Mater. Cycles Waste Manag..

[10] Yang W.S., Park J.K., Park S.W., Seo Y.C. (2015). Past, present and future of waste management in Korea. J. Mater. Cycles Waste Manag..

[11] Huang Q., Wang Q., Dong L., Xi B., Zhou B. (2006). The current situation of solid waste management in China. J. Mater. Cycles Waste Manag..

[12] Narayana T. (2009). Municipal solid waste management in India: From waste disposal to recovery of resources?. Waste Manag..

[13] Shekdar A.V. (2009). Sustainable solid waste management: An integrated approach for Asian countries. Waste Manag..

[14] Young C.Y., Ni S.P., Fan K.S. (2010). Working towards a zero waste environment in Taiwan. Waste Manag. Res..

[15] Song Q., Li J., Zeng X. (2015). Minimizing the increasing solid waste through zero waste strategy. J. Clean. Prod..

[16] Berkel R.V., Fujita T., Hashimoto S., Geng Y. (2009). Industrial and urban symbiosis in Japan: Analysis of the Eco-Town program 1997–2006. J. Environ. Manag..

[17] Tsai W.T. (2020). Recycling of waste electrical & electronic equipment (WEEE) and its toxics-containing management in Taiwan—A case study. Toxics.

[18] Lin C.C., Lin C.H. (2005). What substances or objects should be recycled? The recycling legislative experience in Taiwan. J. Mater. Cycles Waste Manag..

[19] Chen J.S., Huang C.C., Chu P.Y., Lin K.Y. (2007). Engineering characterization of recycled asphalt concrete and aged bitumen mixed recycling agent. J. Mater. Sci..

[20] Lin P.S., Wu T.L., Chang C.W., Chou B.Y. (2011). Effects of recycling agents on aged asphalt binders and reclaimed asphalt concrete. Mater. Struct..

[21] Yang S.H., Lee L.C. (2016). Characterizing the chemical and rheological properties of severely aged reclaimed asphalt pavement materials with high recycling rate. Contr. Build. Struct..

[22] Chen H.J., Huang S.S., Tang C.W., Malek M.A., Ean L.W. (2012). Effect of curing environments on strength, porosity and chloride ingress resistance of blast furnace slag cement concretes: A construction site study. Contr. Build. Struct..

[23] Kuo W.T., Wang H.Y., Shu C.Y. (2014). Engineering properties of cementless concrete produced from GGBFS and recycled desulfurization slag. Contr. Build. Struct..

[24] Ho H.L., Huang R., Hwang L.C., Lin W.T., Hsu H.M. (2018). Waste-based pervious concrete for climate-resilient pavements. Materials.

[25] Al-Hamrani A., Kucukvar M., Alnahhal W., Mahdi E., Onat N.C. (2021). Green concrete for a circular economy: A review on sustainability, durability, and structural properties. Materials.

[26] Environmental Protection Administration (EPA, Taiwan) (2020). Yearbook of Environmental Protection Statistics 2019.

[27] Industrial Waste Reporting and Management Information System (EPA, Taiwan). https://waste.epa.gov.tw/RWD/Statistics/?page=Month1.

[28] Laws and Regulation Retrieving System (Ministry of Justice, Taiwan). https://law.moj.gov.tw/Eng/index.aspx.

[29] Establishment of Environmental Science and Technology Parks (ESTP) (EPA, Taiwan). https://www.epa.gov.tw/eng/EBE8CA1A17AF2F1D.

[30] Rafiq W., Musarat M.A., Altaf M., Napiah M., Sutanto M.H., Alaloul W.S., Javed M.F., Mosavi A. (2021). Life cycle cost analysis comparison of hot mix asphalt and reclaimed asphalt pavement: A case study. Sustainability.

[31] Environmental Science and Technology Parks (Ministry of Economic Affairs, Taiwan). https://investtaiwan.nat.gov.tw/showPagecht449?lang=eng&search=449.

